# The Great Indian Obstetrician and Gynecologist: Padmashri Professor Dr. Krishna Menon (1908-1988)

**DOI:** 10.7759/cureus.87812

**Published:** 2025-07-13

**Authors:** Vaishnavi Rajaraman, Sudha Sumathy

**Affiliations:** 1 Obstetrics and Gynaecology, Amrita Vishwa Vidyapeetham Healthcare Campus, Kochi, IND

**Keywords:** eclampsia, historical vigenette, lytic cocktail, maternal health, medical research, m.k. krishna menon, obstetrician & gynecologist, padmashri awardee

## Abstract

Professor Dr. M.K. Krishna Menon (1908-1988) was a pioneering obstetrician and gynecologist whose lifetime contributions transformed maternal and child healthcare in India. Renowned for his groundbreaking innovations, his most notable achievement was the development of the “lytic cocktail” for eclampsia management, a discovery that dramatically reduced maternal mortality and became a cornerstone in obstetric care globally.

This article revisits his educational and professional milestones, examines his clinical innovations, and highlights his contributions to maternal health, obstetric safety, and academic excellence. His work spanned research in anemia, eclampsia, placenta previa, and perinatal mortality, earning him national and international recognition. Dr. Menon's legacy is a testament to visionary leadership, clinical excellence, and unwavering dedication to women’s health. His lifelong commitment to public health and medical education continues to inspire generations of clinicians and researchers.

## Introduction and background

The annals of Indian obstetrics and gynecology are incomplete without recognizing the monumental influence of Professor Dr. M.K. Krishna Menon. A trailblazer in maternal health, Dr. Menon was a physician, educator, reformer, and advocate whose enduring contributions shaped the very fabric of women's healthcare in India. During an era marked by limited access to quality maternal services and high maternal mortality rates, Dr. Menon brought about transformative change through his innovative approaches and evidence-based practices. He laid down the foundations for clinical excellence and elevated maternal and perinatal care to new heights. His contributions transcended national borders, influencing global discourse in maternal and child health. Dr. Menon embodied a rare combination of clinical brilliance, research acumen, and deep compassion. This article explores his far-reaching impact, leadership legacy, and continued relevance in contemporary obstetrics and gynecology [1,2].

## Review

Early life and education

**Figure 1:**
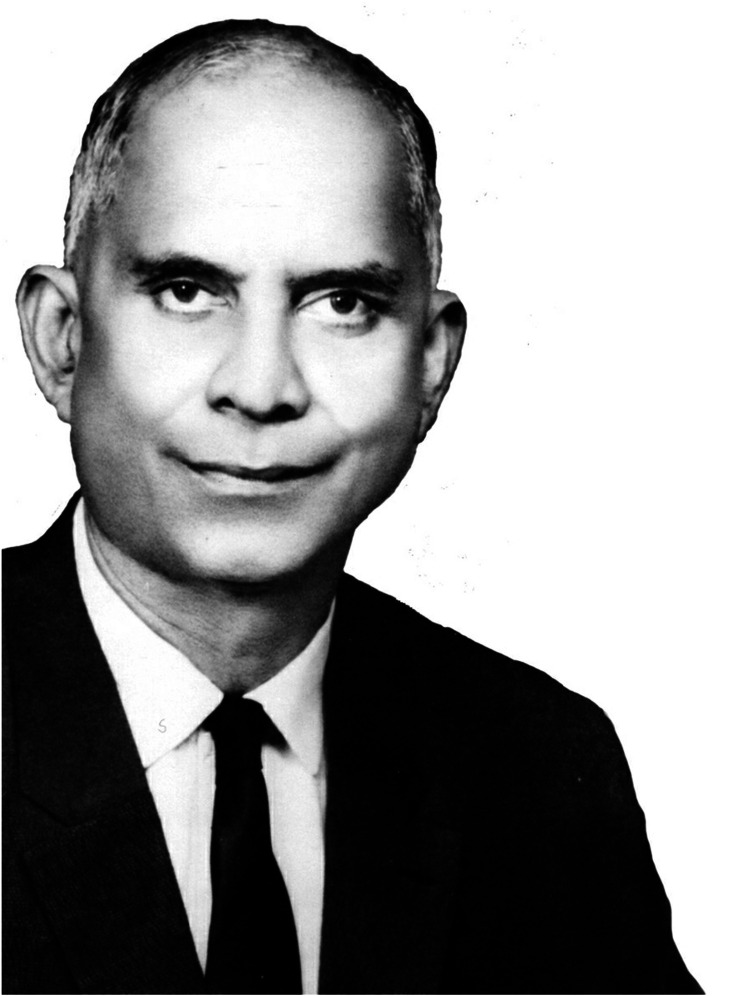
Portrait of Dr. M.K. Krishna Menon Originally published in “Eponyms and Names in Obstetrics and Gynaecology,” Cambridge University Press [3]. License obtained from Cambridge University Press.

Born on January 25, 1908, Dr. Menon was educated at Zamorin’s College, Calicut, Presidency College, and Madras Medical College. He earned his Bachelor of Arts (BA) in 1927, followed by an MBBS (1932), DGO (1935), and MD in Obstetrics and Gynecology (1938), a testament to his academic excellence. His early clinical training took place at the Government General Hospital and the Women and Children’s Hospital, Madras. He later became a Research Scholar at Madras University under the tutelage of Dr. Sir A.L. Mudaliar (Figure 1): Portrait of Dr. M.K. Krishna Menon. Originally published in “Eponyms and Names in Obstetrics and Gynaecology,” Cambridge University Press [1-3].

Career and leadership roles

After his service in the Army Medical Corps during World War II, where he rose to the rank of Lieutenant Colonel, Dr. Menon returned to academia. He held professorial roles at Stanley Medical College, Andhra Medical College, and Madras Medical College. In 1957, he was appointed Director and Professor of the Institute of Obstetrics and Gynecology, Madras Medical College. Under his leadership, the institution became a premier center for maternal and child healthcare and postgraduate training. He was pivotal in establishing a dedicated multi-story maternal and child health building, which remains operational today.

Post-retirement, he served as Visiting Professor at Downstate Medical Center (New York, 1968) and Oxford University (1970). Table 1 shows a timeline of major milestones of Dr. Menon.

**Table 1:** Timeline of major milestones of Dr. Menon

Year	Milestone
1908	Born in Calicut, India
1927	Completed Bachelor of Arts (BA) Degree
1932	Earned MBBS from Madras Medical College
1935	Received Diploma in Gynecology and Obstetrics (DGO)
1938	Awarded MD in Obstetrics and Gynecology
1940-1945	Served in Army Medical Corps during World War II
1957	Appointed Director, Institute of Obstetrics and Gynecology, Madras
1960	Introduced lytic cocktail treatment for eclampsia
1962	Awarded Fellowship of the Royal College of Obstetricians and Gynecologists (FRCOG)
1963	President, All India Congress of Obstetrics and Gynecology (AICOG) (Ahmedabad)
1968	Visiting Professor, State University of New York (SUNY) Downstate Medical Center, New York
1970	Visiting Professor, Oxford University
1973	Honored With Padma Shri by the Government of India
1987	Received Honorary Fellowship, Asia and Oceania Federation of Obstetrics and Gynecology
1988	Passed away, leaving behind a rich legacy

Research

Eclampsia and the Lytic Cocktail

Dr. Menon’s most celebrated contribution was the development of the lytic cocktail (a mix of sedatives to stop convulsions) for eclampsia (seizures in pregnancy) - a combination of pethidine (meperidine) and phenothiazines (chlorpromazine and diethazine) - administered in a schedule that significantly improved maternal outcomes. Presented in London in 1960 after managing over 400 cases, the treatment protocol brought maternal mortality down to 2.2% and became widely adopted across Asia and beyond. This marked a paradigm shift in eclampsia management [4,5].

Anemia in Pregnancy

Recognizing anemia as a public health crisis, Dr. Menon’s research drew attention to the dangerously low hemoglobin levels prevalent among Indian women. He championed iron and folic acid supplementation, linking anemia to higher risks of cardiac failure, hemorrhage, and poor fetal outcomes. His studies helped standardize antenatal iron therapy protocols [6].

Antepartum Hemorrhage and Placenta Previa

Dr. Menon conducted a landmark 33-year study on the management of placenta previa, concluding that caesarean section should be the preferred mode of delivery in cases of complete placenta previa to reduce maternal and perinatal mortality [7].

Perinatal Mortality

In a comprehensive study of 8,877 cases, Dr. Menon analyzed perinatal deaths related to traumatic labor, congenital anomalies, toxemia, and infections. His findings led to enhanced obstetric practices and improved pediatric services, significantly lowering perinatal mortality rates in India during the 1960s [8].

Systematic Review of Public Health Challenges

In his seminal article "A Letter from India," Dr. Menon highlighted systemic healthcare issues: doctor-patient ratio deficiencies, inadequate antenatal care, and postpartum infection risks. He advocated for a community-centric model to improve outcomes, influencing national maternal and child health programs [9].

Academic and global recognition

Dr. Menon’s academic stature was recognized both nationally and internationally. He was appointed to the WHO Expert Committee on Maternal and Child Health, where he contributed to shaping global maternal care standards. At the national level, he chaired the Indian Council of Medical Research’s Subcommittee on Fertility Control, reflecting his pivotal role in reproductive health policy. He held leadership roles such as Vice President of the Asian Federation of Obstetrics and Gynaecology and served as President of FOGSI in 1963. As a Founder Fellow of the National Academy of Medical Sciences, he helped shape India’s academic medicine framework. His contributions were also acknowledged through visiting professorships at SUNY Downstate and the University of Oxford [1,2]. 

He co-authored the widely respected textbook "Clinical Obstetrics" with Dr. A.L. Mudaliar, which became a cornerstone of Indian medical education [10].

Honors and awards

Dr. Menon received numerous accolades (Table 2) [1,2]:

**Table 2:** Awards and accolades of Professor Menon

Year	Awards and accolades
1962	Fellowship of the Royal College of Obstetricians and Gynecologists (FRCOG)
1973	Padma Shri Award
1987	Honorary Fellowship - Asia and Oceania Federation of Obstetrics and Gynecology

These honors reflect not only his academic brilliance but also his dedication to social reform through medicine.

## Conclusions

Padma Shri Professor M.K. Krishna Menon was more than a physician - he was a nation-builder, mentor, and reformist. His clinical innovations, especially in managing eclampsia and maternal anemia, have saved countless lives. A modest, cultured man, Dr. Menon left an indelible legacy through his teachings, writings, and public health initiatives. As we strive to improve maternal and neonatal care, his work remains a timeless blueprint for excellence in women’s health.
